# Evaluation of the Hepatoprotective Properties of Traditional Formulations Based on *Cochlospermum tinctorium* Used in Benin

**DOI:** 10.1155/2024/7753008

**Published:** 2024-08-22

**Authors:** Appolinaire K. Dossa, Jean Robert Klotoé, Victorien Dougnon, Eric Agbodjento, Rémi Akotègnon, Fréjus Ohouko, Manoir Hounkanrin, Kévine Vodounnon, Luc V. C. Brun, Fréderic Loko

**Affiliations:** ^1^ Research Unit in Applied Microbiology and Pharmacology of Natural Substances Research Laboratory in Applied Biology Polytechnic School of Abomey-Calavi University of Abomey-Calavi, Abomey-Calavi, Benin; ^2^ Multidisciplinary Research Laboratory for Technical Education (LARPET) of the National Higher School for Technical Education (ENSET) of Lokossa National University of Science Technology Engineering and Mathematics (UNSTIM), Abomey, Benin; ^3^ Cytology and Anatomy Pathology Laboratory Faculty of Medicine University of Parakou, Parakou, Benin

## Abstract

Hepatic diseases represent a public health problem. Among the approaches to their management is the use of traditional treatments based on the use of medicinal plants. In Benin, several recipes based on *Cochlospermum tinctorium* are used in the treatment of hepatitis without a real scientific basis. This study aimed to evaluate the hepatoprotective effects and acute oral toxicity of 10 of these recipes. The variables studied were the variety of *C. tinctorium* (wild form vs. cultivated form), the species associated with *C. tinctorium* (*Combretum micranthum* vs. *Chromolaena odorata*), and the proportion of *C. tinctorium* in the recipe (1; 4/5; 1/2). The hepatoprotective effect of these extracts at doses of 100, 200, and 400 mg/kg/bw was evaluated in Wistar rats subjected to hepatotoxicity induction through the administration of 5 g/kg of paracetamol. Acute oral toxicity was assessed following the OECD 423 protocol. The results revealed an absence of acute oral toxicity for the 10 recipes. The hepatoprotective tests conducted indicated that the hepatoprotective effect of *C. tinctorium* is dose dependent. The wild variety of *C. tinctorium* had a better hepatoprotective effect than the cultivated one. The association with *C. micranthum* enhances the hepatoprotective effect of *C. tinctorium*, unlike that with *C. odorata*. This study emphasizes that the combination of *C. tinctorium* with *C. micranthum* in the treatment of hepatitis is scientifically justified and it exhibits a dose-dependent hepatoprotective effect.

## 1. Introduction

Chronic liver diseases pose a substantial global health challenge, with liver cirrhosis being the ninth leading cause of mortality in Western nations [1]. These conditions encompass chronic viral hepatitis B and C, alcohol-related liver diseases, nonalcoholic fatty liver disease, and hepatocellular carcinoma, with numerous unresolved challenges. Therapies rooted in Western medical practices frequently demonstrate restricted effectiveness come with potential adverse effects and are often financially out of reach, particularly in developing regions [2].

In the context of viral hepatitis, current therapeutic approaches focus on the sustained suppression of hepatitis B virus (HBV) replication as the primary treatment goal. The preferred treatment involves the prolonged administration of nucleotide reverse transcriptase inhibitors such as entecavir (ETV), tenofovir disoproxil fumarate (TDF), or tenofovir alafenamide (TAF) due to their low risk of resistance [3]. However, achieving the desired outcome of hepatitis B surface antigen (HBsAg) loss, which signifies functional cure, is only realized in approximately 1% of chronic hepatitis B patients treated with existing therapies [4]. Consequently, future treatment approaches should prioritize HBsAg elimination in the management of viral hepatitis [3].

In the pursuit of effective and safe therapies for liver diseases, medicinal plants have surfaced as a promising avenue. Numerous plants have exhibited hepatoprotective qualities. Notably, an extract derived from *Rumex abyssinicus* demonstrated a reduction in CCl4-induced elevation of liver enzyme markers at a dosage of 500 mg/kg [5]. The study examined the hepatoprotective potential of the methanolic extract obtained from the fruit pulp of *Adansonia digitata* L. (100 and 200 mg/kg) against CCl4-induced liver damage in rats. Results indicated a notable reduction (*p* < 0.05) in serum levels of AST, ALT, ALP, and bilirubin, accompanied by fewer abnormalities in the liver tissue when compared to the group treated solely with CCl4 [6]. Moreover, certain plants have exhibited antiviral properties against viral hepatitis. For instance, hyperoside extracted from the ethanolic extract of *Abelmoschus manihot* was observed to reduce secretion of HBsAg and hepatitis B antigen (HBeAg) in Hep G2.2.15 cells. In addition, it inhibited HBV DNA concentration dependently on days 5, 10, and 13. Histopathological examination further validated significant enhancement in hepatocellular architecture [7]. In addition, polyphenols extracted from *Camellia sinensis* (green tea) demonstrated inhibition of HBeAg secretion in a manner dependent on both dosage and time, exhibiting an IC50 value (for HBeAg) of 7.34 *μ*g/mL. Moreover, it notably decreased HBV DNA expression in a dose-dependent manner, with an IC50 of 2.54 *μ*g/mL [8].

In Benin, *Cochlospermum tinctorium* A. Rich. is commonly incorporated into diverse medicinal formulations targeting various health conditions, notably liver diseases [9]. It is utilized in both its wild and cultivated forms, frequently in conjunction with *Chromolaena odorata* and *Combretum micranthum*, particularly for addressing liver disease [10]. The scientific literature has documented the hepatoprotective properties of *Cochlospermum tinctorium* [11, 12]. Currently, there is a lack of scientific evidence regarding the hepatoprotective efficacy and safety/toxicity of combining *Cochlospermum tinctorium* with *Chromolaena odorata* and *Combretum micranthum*. Furthermore, limited research exists on the contrasting biological activities between the wild and cultivated forms of *Cochlospermum tinctorium*. Gathering scientific data on these two varieties is crucial and could inform the selection of appropriate sources for particular medicinal uses. This study aimed to assess the hepatoprotective effects and acute oral toxicity of recipes combining wild and cultivated varieties of *Cochlospermum tinctorium*, *Chromolaena odorata*, and *Combretum micranthum* used in the treatment of liver diseases in Benin.

## 2. Materials and Methods

### 2.1. Ethical Consideration

The research protocol received approval from the Ethics Committee of the Research Unit in Applied Microbiology and Pharmacology of natural substances at the University of Abomey-Calavi in Benin (approval no. 0022/2021/CE/URMAPha/UAC). All procedures were carried out following the guidelines of the National Institute of Health (NIH) for the care and use of laboratory animals.

### 2.2. Plant Material

The botanical specimens comprised leaves of *Chromolaena odorata*, leaves of *Combretum micranthum*, and rhizomes of *Cochlospermum tinctorium* (wild and cultivated varieties). These plant components were authenticated at the National Herbarium of Benin, located at the University of Abomey-Calavi, with the following identifiers: YH 356/HNB for *Combretum micranthum* G. Don, YH621/HNB for *Chromolaena odorata* (L.) R. M. King, and YH 622/HNB for *Cochlospermum tinctorium* ex A. Rich.

### 2.3. Animal Material

Female albino Wistar rats, aged 12 weeks and weighing between 180 and 200 g, were selected as the experimental animal models. These rats were sourced from the animal facility at the Institute of Applied Biomedical Sciences (ISBA). Following a 14-day acclimatization period, the rats were randomly allocated to standard cages, providing them with ad libitum access to water and food. Throughout the study, the rats were housed in an environment maintained at a constant temperature of 22°C, with a regular light/dark cycle of 12 hours each.

### 2.4. Preparation of Extracts

The freshly harvested leaves underwent a thorough washing with distilled water, followed by a drying process in the shade at a controlled temperature of 16°C for a duration of 14 days within the facilities of the Research Unit in Applied Microbiology and Pharmacology of natural substances [13]. Similarly, the rhizomes were initially crushed and then subjected to the same drying conditions. The resulting powders were achieved through grinding using an electric mill (RETSCH SM 2000/1430/Upm/Smf) and subsequently stored in labeled glass vials at room temperature. Following this, different formulations were prepared for each of the three recipes, utilizing proportions of 50%, 80%, and 100% of *C. tinctorium* rhizome powder. These formulations were tailored for both wild and cultivated variants of *C. tinctorium* [10]. The composition of each of the 10 recipes included in the study is detailed in Table 1.

Each formulation underwent the production of a hydroethanolic extract through maceration, utilizing the method outlined by Fanou et al. [14]. This process involved dispersing a mass of 50 grams of powder within a water-ethanol mixture (v/v). The suspension underwent continuous agitation on an automatic shaker for a period of three days at the laboratory's ambient temperature. Following this, the macerate was collected through filtration using hydrophilic cotton and Whatman No. 1 paper, before being evaporated in an oven set at 40°C.

### 2.5. Hepatoprotective Test

The hepatoprotective assay followed a methodology inspired by the research of Ayenew and Wasihun [15] and involved a total of one hundred and sixty-five rats, divided into 33 groups consisting of 5 rats each (Table 2). Among these groups, 3 served as control batches. The first control group (LT) received no treatment. The remaining 32 groups were orally administered different substances for 7 consecutive days: distilled water for the positive control (LTP), silymarin for the reference control (LR), and recipes R1 to R10 at doses of 100, 200, and 400 mg/kg for the 30 test groups. On the 8th day, all 32 groups received a single dose of paracetamol at a dosage of 5 g/kg. 24 hours after paracetamol administration, blood samples were taken from fasted rats anesthetized with thiopental. Thiopental was administered to rats at a dose of 30 mg/kg/body weight by an intravenous route. Following this administration, blood was drawn from the retro-orbital sinus of Wistar rats. Biochemical parameters such as transaminases, direct and total bilirubin, and alkaline phosphatase were evaluated.

A histological study was carried out on the livers removed from the animals after euthanasia with thiopental. Thiopental was administered to Wistar rats intravenously at a dose of 100 mg/kg body weight. After administration, the death of the animals, placed in individual cages, was confirmed by the absence of respiration, heartbeat, or reflexes. In each group, two rats were sacrificed using this method. Following death, the rats were dissected and their livers removed. After removal, the rat body was placed in an animal-type biological waste bag and the appropriate animal carcass disposal procedure was followed. Finally, all equipment was cleaned and disinfected, including cages and work surfaces, and needles and syringes were properly eliminated.

### 2.6. Acute Oral Toxicity

The research adhered to OECD guideline 423 [16] for the acute oral toxicity class assessment. A total of thirty-three female albino Wistar rats were utilized and divided into 11 groups based on their weight (refer to Table 3). The test groups received a single oral dose of 2000 mg/kg simultaneously on the first day (D0) via oral gavage. The control group received distilled water under identical conditions. Each rat in every group was individually marked and closely monitored throughout the 14-day experiment duration. Blood samples were taken from Wistar rats at the start and end of the experiment to assess biochemical and hematological parameters. For each sampling, rats were anesthetized with thiopental. Thiopental was administered to rats at a dose of 30 mg/kg/body weight by an intravenous route. Following this administration, blood was drawn from the retro-orbital sinus of Wistar rats.

### 2.7. Statistical Data Analysis

The statistical analysis was performed using SPSS software version 26.0. Mean values and standard deviations were calculated for each parameter. To assess hepatoprotective effects, univariate analysis of variance was employed to compare data among batches tested at varying doses, as well as between test and control batches (normal, reference, and paracetamol batches). In the toxicity test, the *t*-test was utilized to compare data from the test batches to the normal batch. A significance level of *α* = 0.05 was set for all statistical tests performed.

## 3. Results

### 3.1. Effect of *Cochlospermum tinctorium* Associated with *Chromolaena odorata* and *Combretum micranthum* on the Biochemical Parameters of Liver Function

**Figure 1:**
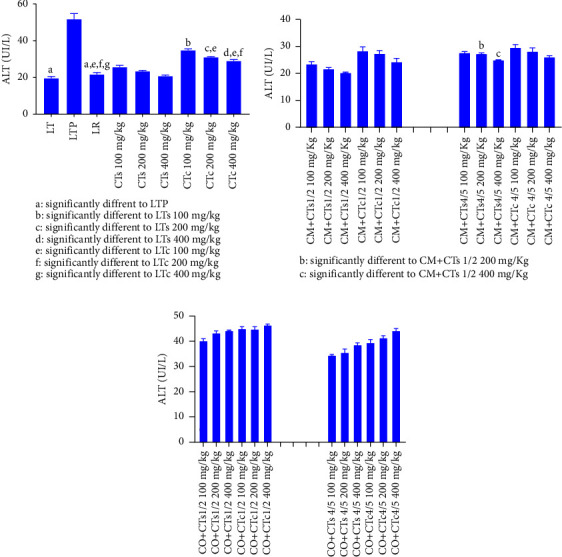
ALT levels of Wistar rats from different batches. (a) Data relating to ALT levels of batches treated with extracts of the wild form of *Cochlospermum tinctorium*, the cultivated form of *Cochlospermum tinctorium*, normal control batch (LT), the reference batch (LR), and the positive control batch treated only with paracetamol (LTP). (b) Data relating to ALT levels of batches treated with extracts from the combination of *Cochlospermum tinctorium* (wild and cultivated forms) with *Combretum micranthum* in 1/2 and 4/5 proportions at different doses. (c) Data relating to ALT levels of batches treated with extracts from the combination of *Cochlospermum tinctorium* (wild and cultivated forms) with *Chromolaena odorata* in 1/2 and 4/5 proportions at different doses. LT: normal control batch; LR: reference batch treated with silymarin; LTP: positive control treated with only paracetamol; CO: *Chromolaena odorata*; CM: *Combretum micranthum*; CTs: *C. tinctorium* (wild) 100%; CTc: *C. tinctorium* (cultivated) 100%; CO + CTs4/5: *C. tinctorium* 80% (wild) and 20% *C. odorata*; CO + CTs1/2: *C. tinctorium* 50% (wild) and 50% *C. odorata*; CO + CTc4/5: *C. tinctorium* 80% (cultivated) and 20% *C. odorata*; CO + CTc1/2: *C. tinctorium* 50% (cultivated) and 50% *C. odorata*; CM + CTs1/2: *C. tinctorium* 50% (wild) and 50% *C. micranthum*; CM + CTs4/5: *C. tinctorium* 80% (wild) and 20% *C. micranthum*; CM + CTc1/2: *C. tinctorium* 50% (cultivated) and 50% *C. micranthum*; CM + CTc4/5: *C. tinctorium* 80% (cultivated) and 20% *C. micranthum*; 100 mg/kg, 200 mg/kg, and 400 mg/kg represent the doses of administration of extracts.

**Figure 2:**
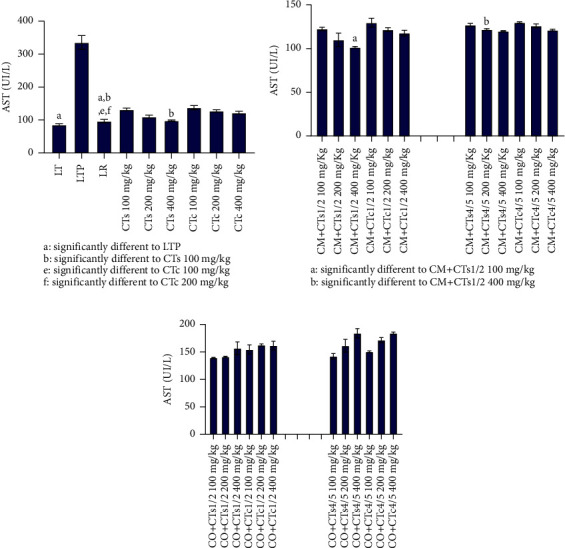
AST levels of Wistar rats from different batches. (a) Data relating to AST levels of batches treated with extracts of the wild form of *Cochlospermum tinctorium*, the cultivated form of *Cochlospermum tinctorium*, normal control batch (LT), the reference batch (LR), and the positive control batch treated only with paracetamol (LTP). (b) Data relating to AST levels of batches treated with extracts from the combination of *Cochlospermum tinctorium* (wild and cultivated forms) with *Combretum micranthum* in 1/2 and 4/5 proportions at different doses. (c) Data relating to AST levels of batches treated with extracts from the combination of *Cochlospermum tinctorium* (wild and cultivated forms) with *Chromolaena odorata* in 1/2 and 4/5 proportions at different doses. LT: normal control batch; LR: reference batch treated with silymarin; LTP: positive control treated with only paracetamol; CO: *Chromolaena odorata*; CM: *Combretum micranthum*; CTs: *C. tinctorium* (wild) 100%; CTc: *C. tinctorium* (cultivated) 100%; CO + CTs4/5: *C. tinctorium* 80% (wild) and 20% *C. odorata*; CO + CTs1/2: *C. tinctorium* 50% (wild) and 50% *C. odorata*; CO + CTc4/5: *C. tinctorium* 80% (cultivated) and 20% *C. odorata*; CO + CTc1/2: *C. tinctorium* 50% (cultivated) and 50% *C. odorata*; CM + CTs1/2: *C. tinctorium* 50% (wild) and 50% *C. micranthum*; CM + CTs4/5: *C. tinctorium* 80% (wild) and 20% *C. micranthum*; CM + CTc1/2: *C. tinctorium* 50% (cultivated) and 50% *C. micranthum*; CM + CTc4/5: *C. tinctorium* 80% (cultivated) and 20% *C. micranthum*; 100 mg/kg, 200 mg/kg, and 400 mg/kg represent the doses of administration of extracts.

**Figure 3:**
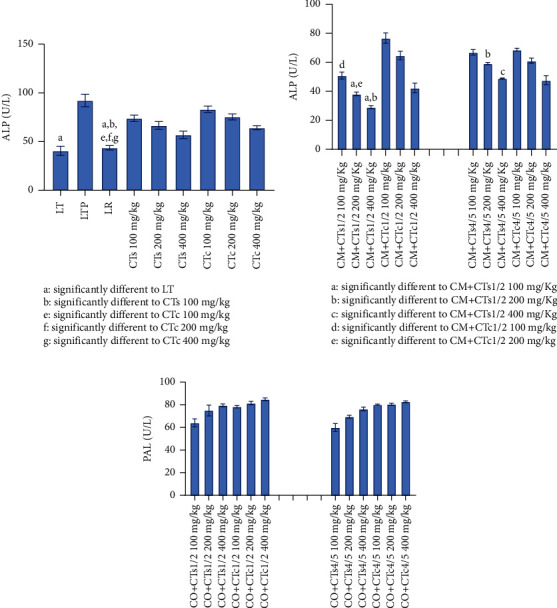
Alkaline phosphatase levels of Wistar rats from different batches. (a) Data relating to alkaline phosphatase levels of batches treated with extracts of the wild form of *Cochlospermum tinctorium*, the cultivated form of *Cochlospermum tinctorium*, normal control batch (LT), the reference batch (LR), and the positive control batch treated only with paracetamol (LTP). (b) Data relating to alkaline phosphatase levels of batches treated with extracts from the combination of *Cochlospermum tinctorium* (wild and cultivated forms) with *Combretum micranthum* in 1/2 and 4/5 proportions at different doses. (c) Data relating to alkaline phosphatase levels of batches treated with extracts from the combination of *Cochlospermum tinctorium* (wild and cultivated forms) with *Chromolaena odorata* in 1/2 and 4/5 proportions at different doses. LT: normal control batch; LR: reference batch treated with silymarin; LTP: positive control treated with only paracetamol; CO: *Chromolaena odorata*; CM: *Combretum micranthum*; CTs: *C. tinctorium* (wild) 100%; CTc: *C. tinctorium* (cultivated) 100%; CO + CTs4/5: *C. tinctorium* 80% (wild) and 20% *C. odorata*; CO + CTs1/2: *C. tinctorium* 50% (wild) and 50% *C. odorata*; CO + CTc4/5: *C. tinctorium* 80% (cultivated) and 20% *C. odorata*; CO + CTc1/2: *C. tinctorium* 50% (cultivated) and 50% *C. odorata*; CM + CTs1/2: *C. tinctorium* 50% (wild) and 50% *C. micranthum*; CM + CTs4/5: *C. tinctorium* 80% (wild) and 20% *C. micranthum*; CM + CTc1/2: *C. tinctorium* 50% (cultivated) and 50% *C. micranthum*; CM + CTc4/5: *C. tinctorium* 80% (cultivated) and 20% *C. micranthum*; 100 mg/kg, 200 mg/kg, and 400 mg/kg represent the doses of administration of extracts.

Figure 1 illustrates the data concerning ALT levels in Wistar rats across various experimental groups. It is apparent from the figure that rats in the paracetamol control group exhibited significantly higher ALT levels (*p* < 0.05) compared to the normal control group, indicating liver function impairment due to paracetamol administration. Conversely, silymarin significantly reduced ALT levels compared to rats receiving extracts from both cultivated and wild forms of *C. tinctorium*. Among the two varieties of *C. tinctorium* tested, the extract from the wild form significantly decreased ALT levels compared to the cultivated form (*p* < 0.05) (Figure 1(a)). Regarding the different combinations of plants composing the multiplant medicinal recipes (Figure 1(b)), the data indicated that the extract derived from an equal combination (50%) of the wild form of *C. tinctorium* and *C. micranthum* significantly reduced ALT levels compared to the combination of 80% *C. tinctorium* and 20% *C. micranthum*. However, concerning the recipe combining *C. tinctorium* and *C. odorata*, rats treated with extracts from different formulations exhibited an increase in ALT levels. This increase was more pronounced for the equal combinations (Figure 1(c)). In contrast, Figure 2 displays data concerning the AST levels of Wistar rats across various groups. Examination of this figure reveals a notable increase in AST levels (*p* < 0.05) in rats treated with paracetamol. Conversely, the group treated with silymarin, serving as a reference compound, demonstrated a significant decrease in AST levels (*p* < 0.05). Similarly, groups treated with extracts of *C. tinctorium* exhibited reduced AST levels. Particularly, the extract derived from the wild form of *C. tinctorium* displayed a significant decrease in AST levels compared to the cultivated form of *C. tinctorium*. Regarding extracts from plant combinations, the extract obtained from the combination of *C. micranthum* and *C. tinctorium* induced a significant reduction in AST at a dose of 400 mg/kg/body weight. From Figure 3, which illustrates the alkaline phosphatase levels of Wistar rats, it becomes apparent that there is a significant increase in the level of this biochemical parameter observed in rats treated solely with paracetamol. Conversely, silymarin administration leads to a notable reduction in alkaline phosphatase levels. In terms of the two forms of *C. tinctorium*, the extract derived from the wild form demonstrates a decrease in the level of this biochemical parameter compared to the cultivated form.

The combination of *C. micranthum* with the wild form of *C. tinctorium* enhances this reduction effect on alkaline phosphatase levels, particularly at doses of 200 and 400 mg/kg/body weight. However, when combined with *C. odorata*, there is an increase in the alkaline phosphatase levels of Wistar rats.

**Figure 4:**
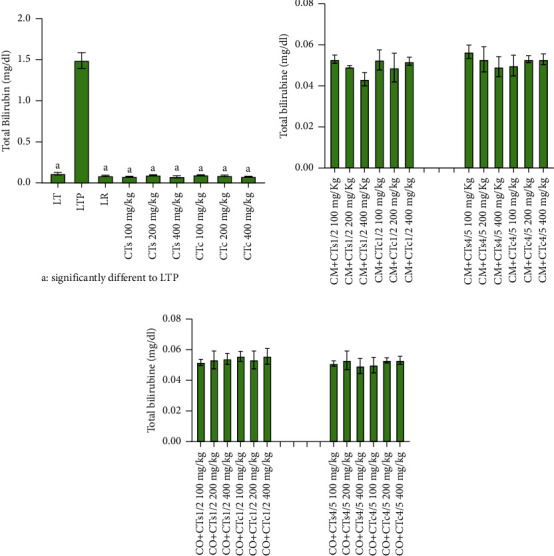
Total bilirubin levels in Wistar rats from different batches. (a) Data relating to total bilirubin levels of batches treated with extracts of the wild form of *Cochlospermum tinctorium*, the cultivated form of *Cochlospermum tinctorium*, normal control batch (LT), the reference batch (LR), and the positive control batch treated only with paracetamol (LTP). (b) Data relating to total bilirubin levels of batches treated with extracts from the combination of *Cochlospermum tinctorium* (wild and cultivated forms) with *Combretum micranthum* in 1/2 and 4/5 proportions at different doses. (c) Data relating to total bilirubin levels of batches treated with extracts from the combination of *Cochlospermum tinctorium* (wild and cultivated forms) with *Chromolaena odorata* in 1/2 and 4/5 proportions at different doses. LT: normal control batch; LR: reference batch treated with silymarin; LTP: positive control treated with only paracetamol; CO: *Chromolaena odorata*; CM: *Combretum micranthum*; CTs: *C. tinctorium* (wild) 100%; CTc: *C. tinctorium* (cultivated) 100%; CO + CTs4/5: *C. tinctorium* 80% (wild) and 20% *C. odorata*; CO + CTs1/2: *C. tinctorium* 50% (wild) and 50% *C. odorata*; CO + CTc4/5: *C. tinctorium* 80% (cultivated) and 20% *C. odorata*; CO + CTc1/2: *C. tinctorium* 50% (cultivated) and 50% *C. odorata*; CM + CTs1/2: *C. tinctorium* 50% (wild) and 50% *C. micranthum*; CM + CTs4/5: *C. tinctorium* 80% (wild) and 20% *C. micranthum*; CM + CTc1/2: *C. tinctorium* 50% (cultivated) and 50% *C. micranthum*; CM + CTc4/5: *C. tinctorium* 80% (cultivated) and 20% *C. micranthum*; 100 mg/kg, 200 mg/kg, and 400 mg/kg represent the doses of administration of extracts.

**Figure 5:**
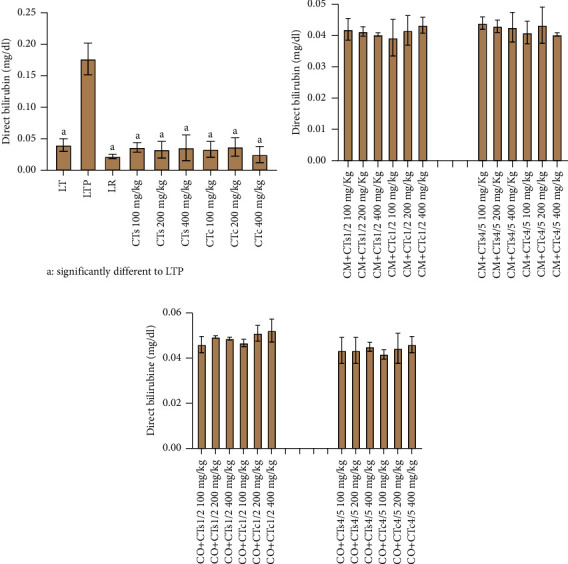
Direct bilirubin levels of Wistar rats from different batches. (a) Data relating to direct bilirubin levels of batches treated with extracts of the wild form of *Cochlospermum tinctorium*, the cultivated form of *Cochlospermum tinctorium*, normal control batch (LT), the reference batch (LR), and the positive control batch treated only with paracetamol (LTP). (b) Data relating to direct bilirubin levels of batches treated with extracts from the combination of *Cochlospermum tinctorium* (wild and cultivated forms) with *Combretum micranthum* in 1/2 and 4/5 proportions at different doses. (c) Data relating to direct bilirubin levels of batches treated with extracts from the combination of *Cochlospermum tinctorium* (wild and cultivated forms) with *Chromolaena odorata* in 1/2 and 4/5 proportions at different doses. LT: normal control batch; LR: reference batch treated with silymarin; LTP: positive control treated with only paracetamol; CO: *Chromolaena odorata*; CM: *Combretum micranthum*; CTs: *C. tinctorium* (wild) 100%; CTc: *C. tinctorium* (cultivated) 100%; CO + CTs4/5: *C. tinctorium* 80% (wild) and 20% *C. odorata*; CO + CTs1/2: *C. tinctorium* 50% (wild) and 50% *C. odorata*; CO + CTc4/5: *C. tinctorium* 80% (cultivated) and 20% *C. odorata*; CO + CTc1/2: *C. tinctorium* 50% (cultivated) and 50% *C. odorata*; CM + CTs1/2: *C. tinctorium* 50% (wild) and 50% *C. micranthum*; CM + CTs4/5: *C. tinctorium* 80% (wild) and 20% *C. micranthum*; CM + CTc1/2: *C. tinctorium* 50% (cultivated) and 50% *C. micranthum*; CM + CTc4/5: *C. tinctorium* 80% (cultivated) and 20% *C. micranthum*; 100 mg/kg, 200 mg/kg, and 400 mg/kg represent the doses of administration of extracts.

Regarding total bilirubin, rats treated with paracetamol alone display an increase in the level of this biochemical parameter (Figure 4). Conversely, administration of silymarin and extracts from both the wild and cultivated forms of 100% *C. tinctorium* induce a reduction in total bilirubin levels. Concerning the combination of *C. micranthum* and *C. odorata* with *C. tinctorium*, no significant influence is noted on the level of this parameter across different groups of rats. The same observations apply to direct bilirubin (Figure 5).

### 3.2. Effect of *Cochlospermum tinctorium* Associated with *Chromolaena odorata* and *Combretum micranthum* on the Histology of Wistar Rat Livers

Table 4 provides details from the histological examination of rat livers across different groups. In the normal control group, liver histology appears normal, with hepatocytes arranged in cords separated by sinusoids (S) around the centrolobular vein. Rats treated solely with paracetamol exhibit discrete congestion, along with the presence of rare isolated hepatocytes displaying eosinophilic cytoplasm and dense pyknotic nuclei. Mild to moderate mixed inflammatory infiltrates, composed of lymphocytes, neutrophilic polymorphonuclear cells, and eosinophilic polymorphonuclear cells, are observed in certain lobules, organized around necrotic cells. In groups treated with wild *C. tinctorium* (100%), a combination of wild *C. tinctorium* 50% and *C. micranthum* 50%, as well as the silymarin reference group, eosinophilic polymorphonuclear cells are noted around the centrolobular veins, along with rare inflammatory infiltrates comprising lymphocytes and eosinophilic polymorphonuclear cells in the portal spaces. The liver retains a subnormal histological architecture.

However, in groups combining *C. tinctorium* with *C. odorata* and all other remaining groups, liver histology displays slight congestion of sinusoids, portal spaces, and centrolobular veins. In addition, rare eosinophilic polymorphonuclear cells are observed within the lobules, along with vacuolar degeneration of hepatocytes.

### 3.3. Acute Oral Toxicity of *Cochlospermum tinctorium* Associated with *Chromolaena odorata* and *Combretum micranthum*

#### 3.3.1. Effect of *Cochlospermum tinctorium* Extracts Alone and in Combination with *Chromolaena odorata* and *Combretum micranthum* on the Biochemical Parameters of Wistar Rats

Table 5 displays data concerning the biochemical parameters of Wistar rats subjected to various treatments. Upon examination of this table, it becomes apparent that, compared to the control group, the groups treated with *C. tinctorium* (alone), regardless of whether it was in the wild or cultivated form, as well as the combinations with *C. odorata* and *C. micranthum*, exhibited no significant differences for each of these parameters (ALT, AST, urea, and creatinine). On the other hand, Table 6 provides information regarding hematological parameters. Analysis of this table indicates that there were no significant differences between the values of the test groups and the control group for each parameter (*p* > 0.05).

Table 7 presents the values of body weight in Wistar rats from various groups. This table highlights a noticeable increase in body weight among rats across all groups. Nevertheless, there was no significant variation observed in the weight gain of Wistar rats among the different groups.

*Mortality of Wistar Rats in Different Groups.* Throughout the 14-day experimental period, no mortality occurred among rats in the various experimental groups. This implies that the LD50 of the tested extracts exceeds 2000 mg/kg/bw.

## 4. Discussion

Liver diseases are a significant global health concern, attributed to various factors including medication use, alcohol consumption, infections (viral, bacterial, or parasitic), and exposure to hepatotoxic agents. In Africa, traditional treatments often involve medicinal plants, emphasizing the importance of evaluating their efficacy. Medicinal plants may contain bioactive compounds with hepatoprotective properties, making them promising candidates for new treatments. This study aimed to assess the hepatoprotective effects and acute oral toxicity of medicinal formulations combining *Cochlospermum tinctorium* with *Chromolaena odorata* on one hand and with *Combretum micranthum* on the other used in the treatment of liver diseases in Benin.

Herbal formulations hold deep cultural significance in the African society, often serving as traditional remedies. With continued reliance on these treatments, it is essential to assess their efficacy and safety to promote proper healthcare practices. In Benin, medicinal blends containing *Cochlospermum tinctorium*, *Chromolaena odorata*, and *Combretum micranthum* are utilized for liver disease treatment without scientific validation. Findings from this study revealed that among the tested *C. tinctorium* varieties, the wild form notably reduced ALT levels compared to the cultivated type (*p* < 0.05) (Figure 1(a)). In addition, a dose-dependent decrease in ALT levels was observed. Similar reports highlighting the hepatoprotective properties of *Cochlospermum tinctorium* have been documented in the literature [17, 18]. This effectiveness can be attributed to the polyphenolic compounds identified in the plant, such as flavonoids and total polyphenols [19].

The variance in hepatoprotective efficacy observed between the wild and cultivated varieties of *Cochlospermum tinctorium* may stem from multiple factors, such as differences in chemical composition, active ingredient content, and growth conditions. Plants often display variations in their chemical makeup depending on environmental factors such as soil quality, climate conditions, sunlight exposure, and nutrient availability. Consequently, the chemical profiles of wild and cultivated forms of *Cochlospermum tinctorium* may diverge, potentially influencing their respective hepatoprotective properties [19]. Furthermore, the wild variant of the plant may encounter a wider array of soil microorganisms in comparison to its cultivated counterpart. This biodiversity can impact the symbiotic relationship between the plant and beneficial microorganisms, which might contribute to the production of health-promoting active metabolites. In addition, wild plants often contend with more adverse environmental conditions, leading to induced stress. In reaction to this stress, wild plants may generate secondary metabolites in varying quantities when compared to cultivated plants [20, 21]. Regarding the various plant combinations utilized in the multiplant medicinal formulations, the data obtained indicate that the extract resulting from an equal (50%) combination of the wild form of *C. tinctorium* and *C. micranthum* significantly maintained normal ALT activity compared to the combination of 80% *C. tinctorium* and 20% *C. micranthum*. These findings may be attributed to an additive effect, considering both plants possess hepatoprotective properties [18, 22]. This phenomenon is associated with microscopic examinations of histological sections, which reveal a reduction in paracetamol-induced hepatic necrosis. Furthermore, Klotoé [19] has demonstrated that combinations of *C. tinctorium* extracts with *C. odorata* and *C. micranthum* are rich in total flavonoids and polyphenols, and phytochemical constituents are known for their role in the hepatoprotective properties of medicinal plants.

In the scientific literature, comparable findings have been documented for a blend of plants, including *Cochlospermum tinctorium*, *Terminalia macroptera*, *Leptadenia hastata*, and *Commiphora africana*, in Burkina Faso [23]. The authors noted that administering this blend of plants to hepatotoxic Wistar rats resulted in a marked decrease in plasma transaminases and alkaline phosphatase levels compared to the negative control group. Histologically treated rats displayed liver tissues that appeared normal or near-normal, varying with the dosage, in contrast to the control group. Likewise, similar findings have been documented for a herbal medicine comprising a standardized combination of three extracts from *Myristica fragrans*, *Astragalus membranaceus*, and *Poria cocos* [24]. The authors noted that this herbal medicine, administered at doses ranging from 150 to 400 mg/kg, exhibited statistically significant dose-dependent suppression of serum alanine aminotransferase (ALT) levels in the acetaminophen model, ranging from 30.8% (*p* ≤ 0.05) to 88.1% (*p*=0.0001), and in the carbon tetrachloride model, ranging from 66.9% (*p*=0.002) to 83.7% (*p*=0.0002), respectively. In addition, this herbal medicine led to reductions of up to 75.7%, 60.9%, and 33.3% in serum levels of aspartate aminotransferase (AST), bile acids, and total bilirubin, respectively. These findings suggest a potential additive effect of *C. tinctorium* and *C. micranthum*. However, in regard to the formulation blending of *C. tinctorium* and *C. odorata*, rats treated with extracts from various formulations did not exhibit normal hepatic marker activity, unlike the combination of *C. tinctorium* and *C. micranthum*, particularly regarding ALT activity. This finding contradicts the anticipated synergistic or additive effect. It is well documented in the scientific literature that *C. odorata* possesses hepatoprotective properties [25, 26]. This observation may be elucidated by factors such as receptor competition, chemical incompatibility, insufficient dosage, pharmacokinetic interactions, or the intricacies of action mechanisms. Active compounds sourced from different plants might engage in competitive inhibition if they target the same liver receptors, or steric hindrance inhibition of active sites if the receptors differ. In instances where two compounds act on the same receptor, one may inhibit or attenuate the effect of the other [27]. Certain chemical compounds found in different plants can interact unfavorably with each other, resulting in an overall decrease in effectiveness. Concerning inadequate dosage, concentrations of active compounds in each plant within the mixture may differ. While one plant may have a high concentration of a specific compound, another plant may contain a lower amount. This variance can result in insufficient dosages of certain compounds, ultimately compromising the overall hepatoprotective effect [28]. Moreover, it is essential to acknowledge that the mechanisms through which plants exert their hepatoprotective effects can be intricated and multifaceted. When two plants target distinct aspects of the hepatoprotective process, their combined effects may not necessarily be synergistic and could even oppose each other. It is crucial to highlight that research on plant interactions and their hepatoprotective effects constitutes a complex and evolving field.

Concerning acute oral toxicity, the findings revealed no instances of mortality or changes in biochemical and hematological parameters. Comparable observations are documented for all the plants tested [29, 30]. This study represents the initial demonstration that combinations of *C. tinctorium* with *C. micranthum*, as well as with *C. odorata*, are devoid of toxicity, as determined by the assessment of hematological and biochemical parameters.

## 5. Conclusion

This study highlighted that the combination of *C. tinctorium* with *C. micranthum* presents a better hepatoprotective effect and is nontoxic. This may justify the use of this medicinal recipe in the traditional Beninese medicine. However, although nontoxic, the combination of C. *tinctorium* with *C. odorata* does not maintain the activity of hepatic markers in Wistar rats making hepatotoxic via paracetamol. Further studies are necessary for more scientific evidence.

## Figures and Tables

**Table 1:** Composition of medicinal recipes.

Recipes	Recipe code	Recipe no.
*C. tinctorium* (wild) 100%	CTs	R1
*C. tinctorium* (cultivated) 100%	CTc	R2
*C. tinctorium* 80% (wild) and 20% *C. odorata*	CO + CTs4/5	R3
*C. tinctorium* 50% (wild) and 50% *C. odorata*	CO + CTs1/2	R4
*C. tinctorium* 80% (cultivated) and 20% *C. odorata*	CO + CTc4/5	R5
*C. tinctorium* 50% (cultivated) and 50% *C. odorata*	CO + CTc1/2	R6
*C. tinctorium* 50% (wild) and 50% *C. micranthum*	CM + CTs1/2	R7
*C. tinctorium* 80% (wild) and 20% *C. micranthum*	CM + CTs4/5	R8
*C. tinctorium* 50% (cultivated) and 50% *C. micranthum*	CM + CTc1/2	R9
*C. tinctorium* 80% (cultivated) and 20% *C. micranthum*	CM + CTc4/5	R10

**Table 2:** Different batches of Wistar rats of the hepatoprotective tests.

No	Batches	Signification of the batches
1	LT	Normal control batch
2	LR	Reference batch treated with silymarin
3	LTP	Positive control treated with only paracetamol
4	CTs 100 mg/kg	*C. tinctorium* (wild) 100% at 100 mg/kg
5	CTs 200 mg/kg	*C. tinctorium* (wild) 100% at 200 mg/kg
6	CTs 400 mg/kg	*C. tinctorium* (wild) 100% at 400 mg/kg
7	CTc 100 mg/kg	*C. tinctorium* (cultivated) 100% at 100 mg/kg
8	CTc 200 mg/kg	*C. tinctorium* (cultivated) 100% at 200 mg/kg
9	CTc 400 mg/kg	*C. tinctorium* (cultivated) 100% at 400 mg/kg
10	CO + CTs4/5 100 mg/kg	*C. tinctorium* 80% (wild) and 20% *C. odorata* at 100 mg/kg
11	CO + CTs4/5 200 mg/kg	*C. tinctorium* 80% (wild) and 20% *C. odorata* at 200 mg/kg
12	CO + CTs4/5 400 mg/kg	*C. tinctorium* 80% (wild) and 20% *C. odorata* at 400 mg/kg
13	CO + CTs1/2 100 mg/kg	*C. tinctorium* 50% (wild) and 50% *C. odorata* at 100 mg/kg
14	CO + CTs1/2 200 mg/kg	*C. tinctorium* 50% (wild) and 50% *C. odorata* at 200 mg/kg
15	CO + CTs1/2 400 mg/kg	*C. tinctorium* 50% (wild) and 50% *C. odorata* at 400 mg/kg
16	CO + CTc4/5 100 mg/kg	*C. tinctorium* 80% (cultivated) and 20% *C. odorata* at 100 mg/kg
17	CO + CTc4/5 200 mg/kg	*C. tinctorium* 80% (cultivated) and 20% *C. odorata* at 200 mg/kg
18	CO + CTc4/5 400 mg/kg	*C. tinctorium* 80% (cultivated) and 20% *C. odorata* at 400 mg/kg
19	CO + CTc1/2 100 mg/kg	*C. tinctorium 50%* (cultivated) and 50% *C. odorata* at 100 mg/kg
20	CO + CTc1/2 200 mg/kg	*C. tinctorium* 50% (cultivated) and 50% *C. odorata* at 200 mg/kg
21	CO + CTc1/2 400 mg/kg	*C. tinctorium* 50% (cultivated) and 50% *C. odorata* at 400 mg/kg
22	CM + CTs1/2 100 mg/kg	*C. tinctorium* 50% (wild) and 50% *C. micranthum* at 100 mg/kg
23	CM + CTs1/2 200 mg/kg	*C. tinctorium* 50% (wild) and 50% *C. micranthum* at 200 mg/kg
24	CM + CTs1/2 400 mg/kg	*C. tinctorium* 50% (wild) and 50% *C. micranthum* at 400 mg/kg
25	CM + CTs4/5 100 mg/kg	*C. tinctorium* 80% (wild) and 20% *C. micranthum* at 100 mg/kg
26	CM + CTs4/5 200 mg/kg	*C. tinctorium* 80% (wild) and 20% *C. micranthum* at 200 mg/kg
27	CM + CTs4/5 400 mg/kg	*C. tinctorium* 80% (wild) and 20% *C. micranthum* at 400 mg/kg
28	CM + CTc1/2 100 mg/kg	*C. tinctorium* 50% (cultivated) and 50% *C. micranthum* at 100 mg/kg
29	CM + CTc1/2 200 mg/kg	*C. tinctorium* 50% (cultivated) and 50% *C. micranthum* at 200 mg/kg
30	CM + CTc1/2 400 mg/kg	*C. tinctorium* 50% (cultivated) and 50% *C. micranthum* at 400 mg/kg
31	CM + CTc4/5 100 mg/kg	*C. tinctorium* 80% (cultivated) and 20% *C. micranthum* at 100 mg/kg
32	CM + CTc4/5 200 mg/kg	*C. tinctorium* 80% (cultivated) and 20% *C. micranthum* at 200 mg/kg
33	CM + CTc4/5 400 mg/kg	*C. tinctorium* 80% (cultivated) and 20% *C. micranthum* at 400 mg/kg

**Table 3:** Composition of Wistar rat groups of the toxicity test.

Types of groups	Group code	Group number
Control group	LT	1
*C. tinctorium* (wild) 100%	CTs	2
*C. tinctorium* (cultivated) 100%	CTc	3
*C. tinctorium* 80% (wild) and 20% *C. odorata*	CO + CTs4/5	4
*C. tinctorium* 50% (wild) and 50% *C. odorata*	CO + CTs1/2	5
*C. tinctorium* 80% (cultivated) and 20% *C. odorata*	CO + CTc4/5	6
*C. tinctorium* 50% (cultivated) and 50% *C. odorata*	CO + CTc1/2	7
*C. tinctorium* 50% (wild) and 50% *C. micranthum*	CM + CTs1/2	8
*C. tinctorium* 80% (wild) and 20% *C. micranthum*	CM + CTs4/5	9
*C. tinctorium* 50% (cultivated) and 50% *C. micranthum*	CM + CTc1/2	10
*C. tinctorium* 80% (cultivated) and 20% *C. micranthum*	CM + CTc4/5	11

**Table 4:** Hepatic histology of Wistar rats from different groups.

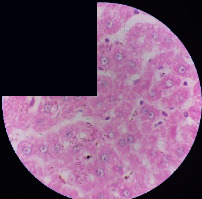 LT	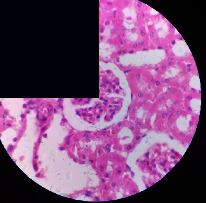 LTP	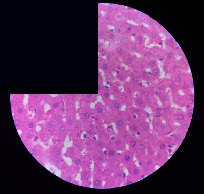 LR	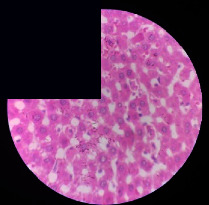 CTs 100 mg/kg

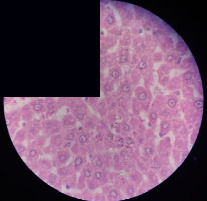 CTs 200 mg/kg	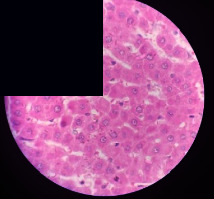 CTs 400 mg/kg	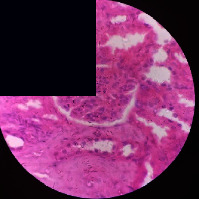 CTc 100 mg/kg	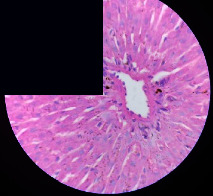 CTc 200 mg/kg

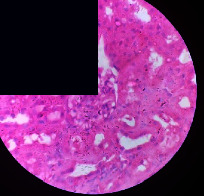 CTc 400 mg/kg	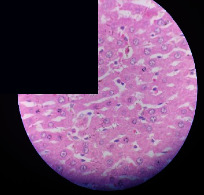 CO + CTs4/5 100 mg/kg	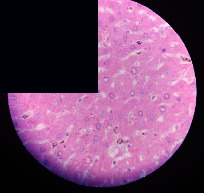 CO + CTs4/5 200 mg/kg	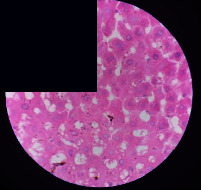 CO + CTs4/5 400 mg/kg

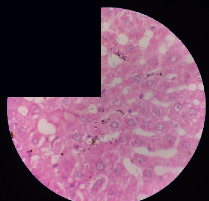 CO + CTs1/2 100 mg/kg	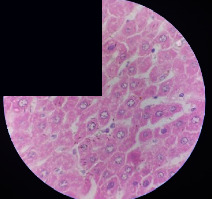 CO + CTs1/2 200 mg/kg	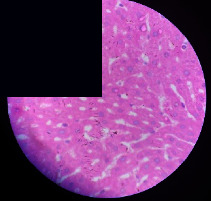 CO + CTs1/2 400 mg/kg	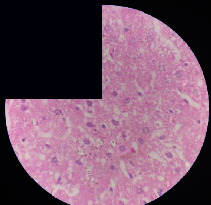 CO + CTc4/5 100 mg/kg

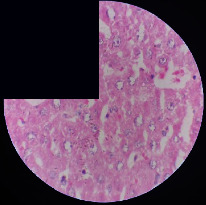 CO + CTc4/5 200 mg/kg	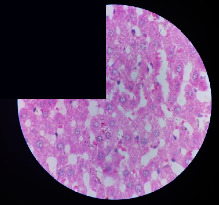 CO + CTc4/5 400 mg/kg	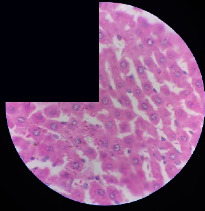 CO + CTc1/2 100 mg/kg	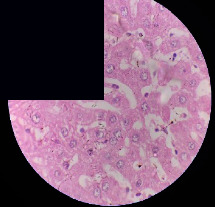 CO + CTc1/2 200 mg/kg

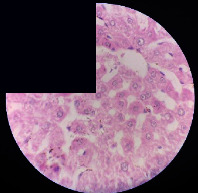 CO + CTc1/2 400 mg/kg	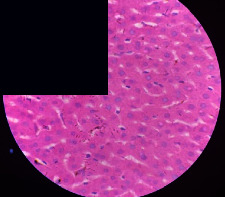 CM + CTs1/2 100 mg/kg	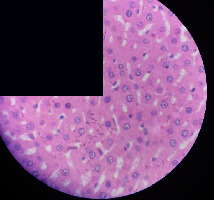 CM + CTs1/2 200 mg/kg	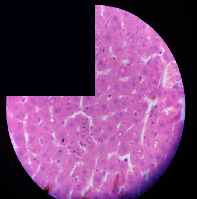 CM + CTs1/2 400 mg/kg

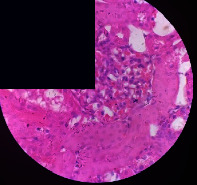 CM + CTs4/5 100 mg/kg	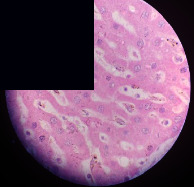 CM + CTs4/5 200 mg/kg	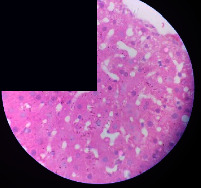 CM + CTs4/5 400 mg/kg	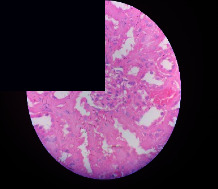 CM + CTc1/2 100 mg/kg

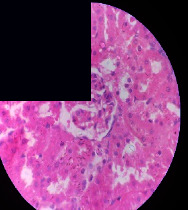 CM + CTc1/2 200 mg/kg	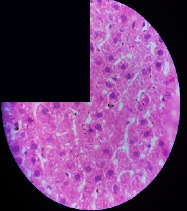 CM + CTc1/2 400 mg/kg	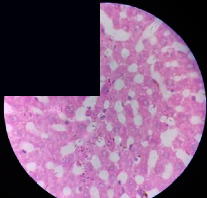 CM + CTc4/5 100 mg/kg	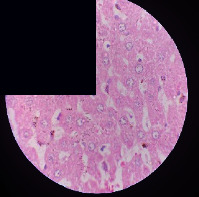 CM + CTc4/5 200 mg/kg

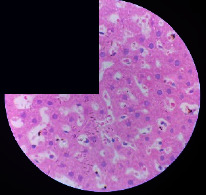 CM + CTc4/5 400 mg/kg			

Histopathological photographs were taken at ×100 magnification. Different batches and constitution of the batches: LT: normal control batch; LR: reference batch treated with silymarin; LTP: positive control treated with only paracetamol; CO: *Chromolaena odorata*; CM: *Combretum micranthum*; CTs: *C. tinctorium* (wild) 100%; CTc: *C. tinctorium* (cultivated) 100%; CO + CTs4/5: *C. tinctorium* 80% (wild) and 20% *C. odorata*; CO + CTs1/2: *C. tinctorium* 50% (wild) and 50% *C. odorata*; CO + CTc4/5: *C. tinctorium* 80% (cultivated) and 20% *C. odorata*; CO + CTc1/2: *C. tinctorium* 50% (cultivated) and 50% *C. odorata*; CM + CTs1/2: *C. tinctorium* 50% (wild) and 50% *C. micranthum*; CM + CTs4/5: *C. tinctorium* 80% (wild) and 20% *C. micranthum*; CM + CTc1/2: *C. tinctorium* 50% (cultivated) and 50% *C. micranthum*; CM + CTc4/5: *C. tinctorium* 80% (cultivated) and 20% *C. micranthum*. 100 mg/kg, 200 mg/kg, and 400 mg/kg represent the doses of administration of extracts. (i) Histological description: LT: in the rats from the normal control group, the histological appearance of the liver is normal. Hepatocytes exhibit a normal appearance and are arranged in cords separated by sinusoids. These sinusoids are arranged around the centrolobular vein. LTP: there is a discreet congestion, presence of rare isolated hepatocytes with eosinophilic cytoplasm and dense pyknotic nuclei, followed by a mixed inflammatory infiltrate of mild to moderate intensity in certain lobules composed of lymphocytes, neutrophilic polymorphonuclear cells, and eosinophilic polymorphonuclear cells. This inflammation is organized around necrotic cells. LR, CTs 100, CTs 200, CTs 400, CM + CTs1/2 100, CM + CTs1/2 200, CM + CTs1/2 400, CM + CTs4/5 100, CM + CTs4/5 200, CM + CTs4/5 400, CM + CTc4/5 100, CM + CTc4/5 200, and CM + CTc4/5 400: the presence of eosinophilic polymorphonuclear cells is observed around the centrolobular veins, along with rare inflammatory infiltrates consisting of lymphocytes and eosinophilic polymorphonuclear cells in the portal spaces. The liver architecture is preserved and thus subnormal. CTc 100, CTc 200, CTc 400, CO + CTc 1/2 100, CO + CTc 1/2 200, CO + CTc 1/2 400, CO + CTc4/5 100, CO + CTc4/5 200, CO + CTc4/5 400, CO + CTs1/2 100, CO + CTs1/2 200, CO + CTs1/2 400, CO + CTs4/5 100, CO + CTs4/5 200, CO + CTs4/5 400, CM + CTc1/2 100, CM + CTc1/2 200, and CM + CTc1/2 400: the histological aspect of the liver is subnormal with slight congestion of sinusoids, portal spaces, and centrolobular veins. There is also the presence of rare eosinophilic polymorphonuclear cells within the lobules and vacuolar degeneration of hepatocytes.

**Table 5:** Values of biochemical parameters in Wistar rats subjected to extracts of *Cochlospermum tinctorium* alone and combined with *Chromolaena odorata* and *Combretum micranthum.*

Batches	Urea (mg/dL)	Standard deviation	Creatinine (mg/dL)	Standard deviation	AST (UI/L)	Standard deviation	(ALT) (UI/L)	Standard deviation
LT	19.32	1.11	0.47	0.02	129.63	4.51	31.58	2.01
CTs	18.01	0.51	0.49	0.03	128.72	3.80	30.91	2.19
CTc	20.26	0.59	0.5	0.02	127.87	4.34	27.07	1.15
CO + CTs4/5	18.63	1.03	0.46	0.04	128.05	0.68	28.69	0.65
CO + CTs1/2	19.55	1.05	0.44	0.02	128.02	3.16	27.55	0.52
CO + CTc4/5	18.52	0.95	0.46	0.01	128.44	2.81	27.66	0.35
CO + CTc1/2	18.92	1.13	0.52	0.03	128.90	0.41	27.70	0.52
CM + CTs1/2	19.15	0.56	0.46	0.01	128.99	0.49	29.77	0.89
CM + CTs4/5	18.63	1.02	0.47	0.02	129.99	0.49	28.44	0.72
CM + CTc1/2	19.58	0.97	0.48	0.03	129.69	0.94	27.55	0.52
CM + CTc4/5	19.64	0.94	0.45	0.03	129.38	1.04	29.91	1.65

**Table 6:** Values of hematological parameters in Wistar rats subjected to extracts of *Cochlospermum tinctorium* alone and combined with *Chromolaena odorata* and *Combretum micranthum*.

Batches	White blood cell	Standard deviation	Red blood cell	Standard deviation	Hemoglobin	Standard deviation	Hematocrit	Standard deviation	MCV	Standard deviation	MCHC	Standard deviation	MCH	Standard deviation	Blood platelets	Standard deviation
LT	3.03	0.50	8.05	0.13	15.27	0.35	44.23	1.44	55.67	1.53	35.60	0.53	36.13	0.68	905.33	45.72
CTs	2.91	0.12	7.88	0.35	14.93	0.38	43.27	1.25	56.33	2.08	35.47	0.72	36.47	1.78	886.33	84.58
CTc	2.80	0.10	8.02	0.04	15.22	0.84	43.01	4.18	55.67	0.58	35.66	0.50	35.03	0.35	897.00	68.50
CO + CTs4/5	4.16	0.51	7.88	0.13	15.40	0.53	43.00	0.66	55.67	0.58	35.59	0.74	36.67	0.90	904.67	35.79
CO + CTs1/2	4.61	0.25	8.04	0.02	15.39	0.61	45.03	0.45	55.27	1.70	35.20	0.82	35.13	1.16	895.73	92.53
CO + CTc4/5	4.13	0.21	8.04	0.06	15.23	0.61	43.93	1.56	55.93	0.40	35.77	0.21	35.47	1.27	869.33	63.16
CO + CTc1/2	4.67	0.10	8.13	0.16	15.57	1.21	45.53	1.46	56.33	0.21	34.57	0.35	34.60	1.15	915.00	38.15
CM + CTs1/2	3.80	0.62	7.98	0.34	15.20	0.35	44.40	1.11	54.96	0.47	35.27	0.50	36.07	0.15	951.00	65.02
CM + CTs4/5	3.64	0.12	8.13	0.15	14.90	0.61	44.20	1.31	55.17	0.55	35.53	0.23	33.67	0.81	891.33	84.97
CM + CTc1/2	3.61	0.16	8.27	0.22	15.17	0.25	43.73	1.29	55.26	0.35	35.97	0.15	33.60	1.13	890.00	11.78
CM + CTc4/5	3.60	0.28	8.16	0.21	14.63	0.58	43.57	2.36	54.83	1.19	35.71	0.32	34.23	1.10	923.33	32.00

**Table 7:** Body weight values of Wistar rats from different groups.

Batches	Time in days	Average (g)	Standard deviation	Weight gain	Standard deviation	Student's *t*-test for weight gain at a 5% threshold
LT	J0	166.67	16.01	6.92	1.86	No significant difference compared to the control group
	J14	178	14			

CTs	J0	164	1	3.25	1.26	No significant difference compared to the control group
	J14	169.33	2.52			

CTc	J0	155.33	2.31	6.89	2.68	No significant difference compared to the control group
	J14	166	2.65			

CO + CTs4/5	J0	156.67	1.53	9.58	2.57	No significant difference compared to the control group
	J14	171.67	3.79			

CO + CTs1/2	J0	163.33	4.17	8.68	6.65	No significant difference compared to the control group
	J14	177.33	6.81			

CO + CTc4/5	J0	163.33	6.429	2.48	1.33	No significant difference compared to the control group
	J14	167.33	4.62			

CO + CTc1/2	J0	160	2	2.92	144	No significant difference compared to the control group
	J14	164.67	3.06			

CM + CTs1/2	J0	160.67	8.08	5.16	2.95	No significant difference compared to the control group
	J14	169	10.54			

CM + CTs4/5	J0	160	7.55	6.59	4.56	No significant difference compared to the control group
	J14	170.33	2.52			

CM + CTc1/2	J0	158.67	1.53	7.76	3.04	No significant difference compared to the control group
	J14	171	6.08			

CM + CTc4/5	J0	168.67	3.06	6.31	2.59	No significant difference compared to the control group
	J14	179.33	6.81			

## Data Availability

The data used to support the findings of this study are included within the published article.
